# CHINESE AMERICAN FAMILY CAREGIVERS’ PERCEPTIONS OF HOME CARE AND FACILITY CARE FOR LOVED ONES WITH ADRD

**DOI:** 10.1093/geroni/igad104.2546

**Published:** 2023-12-21

**Authors:** Chien-Ching Li, Olimpia Paun, Xiaojun Gao, Xiao Ying Lei

**Affiliations:** Rush University, Chicago, Illinois, United States; Rush University, Chicago, Illinois, United States; Rush University Medical Center, Chicago, Illinois, United States; Rush University, Chicago, Illinois, United States

## Abstract

Cognitive decline and impairment among older adults are significant public health concerns because they lead to increased disability, caregiver burden, and healthcare costs associated with the treatment of declining cognitive function. The Chinese American population, the largest Asian group in the US, is aging rapidly, and it is expected that older Chinese Americans and their caregivers will increasingly need ADRD care planning for home care and facility care. Therefore, the goal of this study is to examine the beliefs and attitudes of Chinese American family caregivers regarding home care and facility care for their loved ones with ADRD. In our study, N=17 Chinese American caregivers were recruited to participate in the study interview through a partnership with the Alzheimer’s Program at the Chinese American Service League. The mean age of the caregivers was 49.6 years, and the majority were female, married, had college degrees or higher, and reported being in good health. Results indicated that most ADRD patients needed medium or high levels of care and were cared for at home. Chinese American caregivers were reluctant to place their loved ones in facilities, such as nursing homes or memory care centers, due to high costs, poor quality of care, language barriers, and Chinese filial piety. Additionally, most Chinese caregivers lacked knowledge regarding the types of home care and facility care and the services provided. Therefore, future development of culturally and linguistically targeted interventions for ADRD care planning is necessary for Chinese American patients with ADRD and family caregivers.

